# Diversity of *Colletotrichum* Species Causing Apple Bitter Rot and Glomerella Leaf Spot in China

**DOI:** 10.3390/jof8070740

**Published:** 2022-07-18

**Authors:** Yang Chen, Dandan Fu, Wei Wang, Mark L. Gleason, Rong Zhang, Xiaofei Liang, Guangyu Sun

**Affiliations:** 1 State Key Laboratory of Crop Stress Biology in Arid Areas and College of Plant Protection, Northwest A&F University, Yangling, Xianyang 712100, China; ischenyang@outlook.com (Y.C.); fudandanly@126.com (D.F.); weiwei10210021@126.com (W.W.); rongzh@nwsuaf.edu.cn (R.Z.); 2 College of Food & Bioengineering, Henan University of Science and Technology, Luoyang 471003, China; 3 Department of Plant Pathology and Microbiology, Iowa State University, Ames, IA 50011, USA; mgleason@iastate.edu

**Keywords:** apple bitter rot, *Colletotrichum*, Glomerella leaf spot, *Malus*

## Abstract

Bitter rot and Glomerella leaf spot (GLS) of apples, caused by *Colletotrichum* species, are major diseases of apples around the world. A total of 98 isolates were obtained from apple fruits with bitter rot, and 53 isolates were obtained from leaves with leaf spot in the primary apple production regions in China. These isolates were characterized morphologically, and five gene regions (*ITS*, *ACT*, *GAPDH*, *CHS*-1 and *TUB*2) were sequenced for each isolate. A phylogenetic analysis, combined with a comparison of the morphological, cultural and pathogenic characters, sorted bitter rot isolates into six species: *C. alienum*, *C. fructicola*, *C. gloeosporioides* sensu stricto, *C. nymphaeae*, *C. siamense* and one new species, *C. orientalis* Dandan Fu & G.Y. Sun. Among these, *C. siamense* was the predominant pathogen associated with bitter rot. Isolates from leaf spot were identified as two species, *C. aenigma* and *C. fructicola*. This is the first report of *C. orientalis* as an apple bitter rot pathogen worldwide, and the results provide important insights into the diversity of *Colletotrichum* species in China.

## 1. Introduction

Apple bitter rot (ABR) is a common pre- and post-harvest disease in nearly all apple-growing areas worldwide. Because of its latent infection ability, crop losses can be severe from mid- to late-summer under prolonged warm and wet weather conditions [1]. The earliest record of a pathogen causing ABR is from 1856 when *Gloeosporium fructigenum* was described as the causal agent [2]. The fungus causing ABR was renamed several times until all species became synonymous to *Glomerella cingulata* (Stoneman) Spauld. & H. Schrenk (anamorph: *Colletotrichum gloeosporioides* (Penz.) Penz. & Sacc) in 1903. In 1965, *C. acutatum* J. H. Simmonds was distinguished from *C. gloeosporioides* based on physiology and morphology [3]. ABR pathogens were mainly reported to be *C. gloeosporioides, G. cingulata* and *C. acutatum* [4]. Jones et al. found that *C. acutatum* and *C. gloeosporioides* were recovered from 81% and 19%, respectively, of 165 symptomatic fruits collected from orchards in western Michigan [5]. Shi et al. reported that *C. acutatum* was the most predominant species (70%) associated with ABR in orchards in Arkansas, North Carolina and Virginia [6]. Restriction fragment length polymorphisms (RFLP) and random amplified polymorphic DNA (RAPD) analyses indicated high intraspecific diversity [1,7,8,9], which, however, might reflect interspecific differences in the revised *Colletotrichum* taxonomic system [10]. In addition to apple fruit bitter rot, *Colletotrichum* species also incur foliar disease, namely Glomerella leaf spot (GLS) [11]. GLS was first reported in Brazil in the 1980s and was subsequently reported in the USA and East Asia [1,7]. The disease causes severe leaf fall off on susceptible cultivars, such as Gala and Golden Delicious. The new *Colletotrichum* taxonomic system was established with polyphasic approaches with an emphasis on multigene phylogeny, in which ‘*C. gloeosporioides’* and ‘*C. acutatum*’ are both monophyletic species complexes, with over 20 and 30 independent species, respectively [10,12]. Thus far, a number of ABR pathogenic species, belonging either to the *C. acutatum* species complex (CASC) (*C. abscissum*, *C. acutatum*, *C. fioriniae*, *C. godetiae*, *C. melonis*, *C. nymphaeae* and *C. paranaense*) or the *C. gloeosporioides* species complex (CGSC) (*C. chrysophilum*, *C. fragariae*, *C. fructicola*, *C. gloeosporioides* s. str., *C. noveboracense*, *C. siamense*, *C. alienum* and *C. theobromicola*) have been reported worldwide [13,14,15,16,17,18,19,20,21,22,23,24,25,26,27]. Compared with ABR, relatively few GLS pathogens have been recognized thus far; these include *C. fructicola* and *C. aenigma*, belonging to the CGSC; *C. karstii*, belonging to the *C. boninense* species complex (CBSC); and *C. limetticola*, belonging to the CASC [11,20,28,29,30,31,32].

In China, apple bitter rot occurs in almost all producing areas, and the pathogens have been identified as *C. gloeosporioides* and *C. acutatum* [33,34,35]. Unfortunately, these species may all represent species complexes. GLS is an emerging disease that was first reported in 2012, and the pathogens have been identified as *C. fructicola* and *C. aenigma* [28], yet hidden pathogen diversity may exist due to insufficient investigation. Therefore, the main objective of this study is to investigate the *Colletotrichum* species diversity associated with ABR and GLS in China; gaining this knowledge will provide clues towards more effective control measures against these devastating diseases.

## 2. Materials and Methods

### 2.1. Isolates

Isolates were collected from diseased apple tissues exhibiting bitter rot and leaf spot symptoms in commercial apple orchards in four provinces, including Liaoning, Shandong, Henan and Shaanxi, of China from 2009 to 2013. Small pieces of symptomatic tissue were cut from lesions, immersed in 70% alcohol for 1 min, rinsed with sterile water and then dried on sterilized filter paper before placement into Petri dishes with Potato Dextrose Agar (PDA, Becton, Dickinson and Company, Franklin Lakes, NJ, USA). Cultures were incubated for 4 days at 25 °C in darkness. A mycelial disc was taken from the actively growing edge of a mono-conidial colony, and then transferred onto new PDA plates. Monosporic isolates were obtained from the new cultures. The surfaces of the PDA plates were scraped with sterile water and collected as conidia suspensions. Monosporic isolates were deposited in the Fungal Laboratory of Northwest A&F University, Yangling, Shaanxi Province, China. After 7 days at 25 °C in darkness, the sizes and shapes of 50 conidia harvested from the cultures were measured and recorded [36]. The colony diameter, color of the conidial masses and zonation of the colony were recorded. Appressoria were induced using a slide culture technique, in which a 1 cm^2^ segment of PDA containing the isolate was placed in sterile water in a sterile Petri dish, covered with a sterile coverslip and incubated under high humidity at 25 °C in darkness. After 2 days, the shapes and sizes of 50 appressoria on the coverslip were recorded.

### 2.2. DNA Extraction and PCR Amplification

The protocol from Barnes et al. was used to extract DNA from the mycelia by scraping the surface of the PDA after it had been cultured for 7 days at 25 °C [37]. The quantity and quality of the DNA were estimated by UV microscopic spectrophotometer (Nanodrop 2000, Thermo Fisher Scientific, Waltham, MA, USA). The partial rDNA-*ITS*, actin (*ACT*), glyceraldehyde-3-phosphate dehydrogenase (*GAPDH*), chitin synthase (*CHS*-1) and β-tubulin-2 (*TUB*2) genes were amplified by PCR using primer pairs of ITS1-F [38] + ITS4 [39], ACT-512F + ACT-783R [40], GDF1 + GDR1 [41], CHS-79F + CHS-354R [40] and Bt2a + Bt2b [42], respectively. The PCR protocols were performed as described by Damm et al. [43]. The sequences of the isolates described in this study were deposited in GenBank; the accession numbers are listed in Table 1.

### 2.3. Sequence Alignment and Phylogenetic Analysis

**Figure 1:**
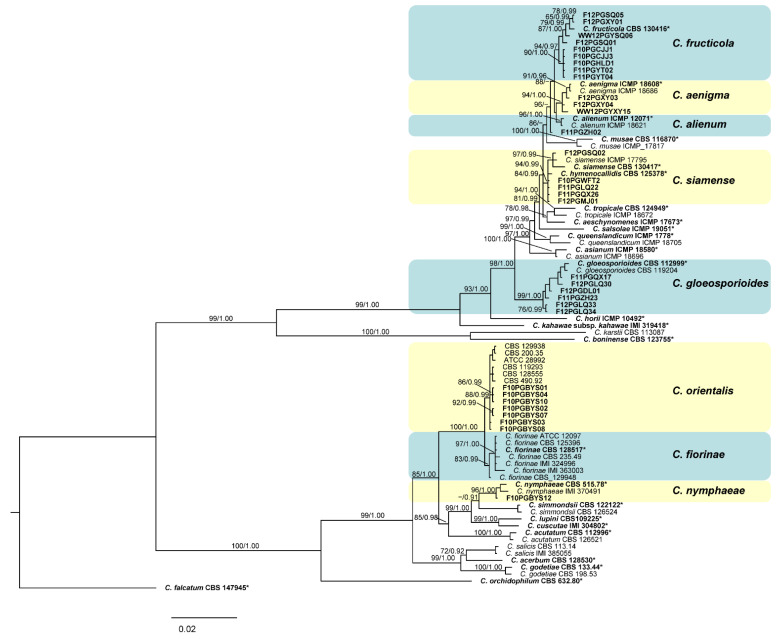
Phylogram of the *Colletotrichum* species resulting from maximum likelihood and Bayesian analyses based on the combined alignment dataset of *ITS*, *ACT*, *GAPDH*, *CHS*-1 and *TUB*-2 sequences. Bootstrap support values above 60% and Bayesian posterior probability values above 0.9 were given at the nodes. Isolates isolated in this study and type strains are shown in bold. * Ex-holotype, ex-neotype, ex-epitype strains.

Preliminary alignments of the multi-locus sequences were conducted using Clustal X [44] with a manual adjustment and BioEdit for visual improvement wherever necessary. The concatenation of the five-gene sequences was completed in PhyloSuite [45]. A maximum likelihood (ML) analysis was performed by RAxML version 8 [46] under the GTR model [47], and a non-parametric bootstrap analysis with 1000 repetitions [48] was used to determine the statistical support of the phylogeny. Bayesian inference (BI) phylogeny construction was performed with MrBayes version 3.2.1 [49], with the GTR + G + I nucleotide substitution model. The analysis included two separate runs for 1 × 10^7^ generations; each run was sampled every 1000 generations, and the convergence of all the parameters was checked using internal diagnostics. To construct the 50% majority-rule consensus tree, the first 25% generations were discarded as burn-in. The phylogenetic tree (Figure 1) was visualized using FigTree v 1.4.4. A potential recombination event between *C. fioriniae* and *C. orientalis* was detected based on a pairwise homoplasy index (PHI) analysis of the Genealogical Concordance Phylogenetic Species Recognition concept in SplitsTree version 4.11.3 using the multi-locus alignment dataset [50,51].

### 2.4. Pathogenicity Tests

Twelve representative isolates of *Colletotrichum* were chosen based on species identity and locations. Healthy apple fruits and leaves were selected, washed with tap water, blown dry in the hood and surface-sterilized with 70% ethanol prior to inoculation. Leaves and fruits were drop-inoculated with the conidia suspension (approximately 10^6^/mL in concentration) or mycelial plugs. The fruits’ wounds were made by sterile insect needles with about 10 holes within a circular area of 5 mm in diameter. After inoculation, the fruits were incubated at 25 °C in plastic bags. The disease incidence of each fungal isolate was recorded 3 days after inoculation. For each isolate, at least five fruit/leaf inoculation replicates were performed in each experiment, and the inoculation experiment was repeated two times.

**Table 1:** Fungal isolates and sequences used in the phylogenetic analysis of this study.

Species	Type Strain	Host	County	GenBank No.				
				*ITS*	*ACT*	*GAPDH*	*CHS*-1	*TUB*2
*C. acerbum*	CBS 128530 ^1^	*Malus domestica*	New Zealand	JQ948459	JQ949780	JQ948790	JQ949120	JQ950110
*C. acutatum*	CBS 112996 ^1^	*Carica papaya*	Australia	JQ005776	JQ005839	JQ948677	JQ005797	JQ005860
	CBS 126521 ^2^	*Anemone* *hybride*	Netherlands	JQ948366	JQ949687	JQ948697	JQ949027	JQ950017
*C. aenigma*	ICMP 18608 ^1^	*Persea americana*	Israel	JX010244	JX009443	JX010044	JX009774	JX010389
	ICMP 18686 ^2^	*Pyrus pyrifolia*	Japan	JX010243	JX009519	JX009913	JX009789	JX010390
	F12PGXY03	*Malus domestica*	China	KF772117	KF772027	KF772087	KF772057	KF772147
	F12PGXY04	*Malus domestica*	China	KF772118	KF772028	KF772088	KF772058	KF772148
	W12PGYXY15	*Malus domestica*	China	KF791590	KF791569	KF791583	KF791576	KF791597
*C. aeschynomenes*	ICMP 17673 ^1^	*Aeschynomene* sp.	USA	JX010176	JX009483	JX009930	JX009799	JX010392
*C. alienum*	ICMP 12071 ^1^	*Malus domestica*	New Zealand	JX010251	JX009572	JX010028	JX009882	JX010411
	ICMP 18621 ^3^	*Persea americana*	New Zealand	JX010246	JX009552	JX009959	JX009755	JX010386
	F11PGZH02	*Malus domestica*	China	KF772119	KF772029	KF772089	KF772059	KF772149
*C. asianum*	ICMP 18580 ^1^	*Coffea arabica*	Thailand	FJ972612	JX009584	JX010053	JX009867	JX010406
	ICMP 18696 ^3^	*Mangifera indica*	Australia	JX010192	JX009576	JX009915	JX009753	JX010384
*C. boninense*	CBS 123755 ^1^	*Crinum asiaticum*	Japan	JQ005153	JQ005501	JQ005240	JQ005327	JQ005588
*C. cuscutae*	IMI 304802 ^1^	*Cuscuta* sp.	Dominica	JQ948195	JQ949516	JQ948525	JQ948856	JQ949846
*C. fioriniae*	CBS 128517 ^1^	*Fiorinia externa*	USA	JQ948292	JQ949613	JQ948622	JQ948953	JQ949943
	CBS 125396 ^2^	*Malus domestica*	USA	JQ948299	JQ949620	JQ948629	JQ948960	JQ949950
	CBS 128517 ^1^	*Fiorinia externa*	USA	JQ948292	JQ949613	JQ948622	JQ948953	JQ949943
	CBS 125396 ^2^	*Malus domestica*	USA	JQ948299	JQ949620	JQ948629	JQ948960	JQ949950
	CBS 363003 ^2^	*Camellia reticulata*	China	JQ948339	JQ949660	JQ948669	JQ949000	JQ949990
	ATCC 28992 ^2^	*Malus domestica*	USA	JQ948297	JQ949618	JQ948627	JQ948958	JQ949948
	CBS 129938 ^2^	*Malus domestica*	USA	JQ948296	JQ949617	JQ948626	JQ948957	JQ949947
	CBS 129948 ^2^	*Tulipa* sp.	UK	JQ948344	JQ949665	JQ948674	JQ949005	JQ949995
	IMI 324996 ^2^	*Malus pumila*	USA	JQ948301	JQ949622	JQ948631	JQ948962	JQ949952
	ATCC 12097 ^2^	*Rhododendron* sp.	USA	JQ948307	JQ949628	JQ948637	JQ948968	JQ949958
	CBS 200.35 ^2^	*Rubus* sp.	USA	JQ948293	JQ949614	JQ948623	JQ948954	JQ949944
	CBS 490.92 ^2^	*Solanum lycopersicum*	New Zealand	JQ948326	JQ949647	JQ948656	JQ948987	JQ949977
	CBS 119293 ^2^	*Vaccinium corymbosum* (blueberry)	New Zealand	JQ948314	JQ949635	JQ948644	JQ948975	JQ949965
*C. fructicola*	CBS 130416 ^1^	*Coffea arabica*	Thailand	JX010165	FJ907426	JX010033	JX009866	JX010405
	F12PGSQ01	*Malus domestica*	China	KF772124	KF772034	KF772094	KF772064	KF772154
	F12PGSQ05	*Malus domestica*	China	KF772125	KF772035	KF772095	KF772065	KF772155
	F12PGXY01	*Malus* ×*domestica*	China	KF772126	KF772036	KF772096	KF772066	KF772156
	W12PGYSQ06	*M.* ×*domestica*	China	KF791591	KF791570	KF791584	KF791577	KF791598
	F10PGCJJ1	*M.* ×*domestica*	China	KF772128	KF772038	KF772098	KF772068	KF772158
	F10PGCJJ3	*M.* ×*domestica*	China	KF772129	KF772039	KF772099	KF772069	KF772159
	F10PGHLD1	*M.* ×*domestica*	China	KF772130	KF772040	KF772100	KF772070	KF772160
	F11PGYT02	*M.* ×*domestica*	China	KF772131	KF772041	KF772101	KF772071	KF772161
	F11PGYT04	*M.* ×*domestica*	China	KF772132	KF772042	KF772102	KF772072	KF772162
*C. gloeosporioides*	CBS 112999 ^1^	*Citrus sinensis*	Italy	JX010152	JX009531	JX010056	JX009818	JX010445
	CBS 119204 ^3^	*Pueraria lobata*	USA	JX010150	JX009502	JX010013	JX009790	GQ849434
	F11PGQX17	*M.* ×*domestica*	China	KF772111	KF772021	KF772081	KF772051	KF772141
	F12PGDL01	*M.* ×*domestica*	China	KF772112	KF772022	KF772082	KF772052	KF772142
	F12PGLQ30	*M.* ××*domestica*	China	KF772113	KF772023	KF772083	KF772053	KF772143
	F12PGLQ33	*M.* ×*domestica*	China	KF772114	KF772024	KF772084	KF772054	KF772144
	F12PGLQ34	*M.* ×*domestica*	China	KF772115	KF772025	KF772085	KF772055	KF772145
	F11PGZH23	*M.* ×*domestica*	China	KF772116	KF772026	KF772086	KF772056	KF772146
*C. godetiae*	CBS 133.44 ^1^	*Clarkia hybrida*	Denmark	JQ948402	JQ949723	JQ948733	JQ949063	JQ950053
	CBS 198.53 ^2^	*M. sylvestris*	Netherlands	JQ948432	JQ949753	JQ948763	JQ949093	JQ950083
*C. horii*	ICPM 10492 ^1^	*Diospyros kaki*	Japan	GQ329690	JX009438	GQ329681	JX009752	JX010450
*C. hymenocallidis*	CBS 125378 ^1^	*Hymenocallis*	China	JX010278	GQ856775	JX010019	GQ856730	JX010410
*C. kahawae* subsp. *kahawae*	IMI 319418 ^1^	*Coffea arabica*	Kenya	JX010231	JX009452	JX010012	JX009813	JX010444
*C. karstii*	CBS 132134 ^4^	*Malus* sp.	USA	JQ005181	JQ005529	JQ005268	JQ005355	JQ005615
*C. lupini*	CBS 109225 ^1^	*Lupinus albus*	Ukraine	JQ948155	JQ949476	JQ948485	JQ948816	JQ949806
*C. musae*	CBS 116870 ^1^	*Musa* sp.	USA	JX010146	JX009433	JX010050	JX009896	HQ596280
	ICMP 17817 ^3^	*Musa sapientum*	Kenya	JX010142	JX009432	JX010015	JX009815	JX010395
*C. nymphaeae*	CBS 515.78 ^1^	*Nymphaea alba*	Netherlands	JQ948197	JQ949518	JQ948527	JQ948858	JQ949848
	IMI 370491 ^2^	*M. pumila*	Brazil	JQ948204	JQ949525	JQ948534	JQ948865	JQ949855
	F10PGBYS12	*M.* ×*domestica*	China	KF772133	KF772043	KF772103	KF772073	KF772163
*C. orchidophilum*	CBS 632.80 ^1^	*Dendrobium* sp.	USA	JQ948151	JQ949472	JQ948481	JQ948812	JQ949802
*C. orientalis*	F10PGBYS1	*M.* ×*domestica*	China	KF772134	KF772044	KF772104	KF772074	KF772164
	F10PGBYS2	*M.* ×*domestica*	China	KF772135	KF772045	KF772105	KF772075	KF772165
	F10PGBYS3	*M.* ×*domestica*	China	KF772136	KF772046	KF772106	KF772076	KF772166
	F10PGBYS4	*M.* ×*domestica*	China	KF772137	KF772047	KF772107	KF772077	KF772167
	F10PGBYS7	*M.* ×*domestica*	China	KF772138	KF772048	KF772108	KF772078	KF772168
	F10PGBYS8	*M.* ×*domestica*	China	KF772139	KF772049	KF772109	KF772079	KF772169
	F10PGBYS10	*M.* ×*domestica*	China	KF772140	KF772050	KF772110	KF772080	KF772170
	CBS 128555 ^2^	*Mal* *us domestica*	New Zealand	JQ948305	JQ949626	JQ948635	JQ948966	JQ949956
*C. queenslandicum*	ICMP 1778 ^1^	*Carica papaya*	Australia	JX010276	JX009447	JX009934	JX009899	JX010414
	ICMP 18705 ^3^	*Coffea* sp.	Fiji	JX010185	JX009490	JX010036	JX009890	JX010412
*C. salicis*	CBS 113.14 ^2^	*M.* ×*domestica*	Germany	JQ948478	JQ949799	JQ948809	JQ949139	JQ950129
	IMI 385055 ^2^	*M.* ×*domestica*	New Zealand	JQ948472	JQ949793	JQ948803	JQ949133	JQ950123
*C. salsolae*	ICMP 19051 ^1^	*Salsola tragus*	Hungary	JX010242	JX009562	JX009916	JX009863	JX010403
*C. siamense*	CBS 130417 ^1^	*Coffea arabica*	Thailand	JX010171	FJ907423	JX009924	JX009865	JX010404
	ICMP 17795 ^3^	*M.* ×*domestica*	USA	JX010162	JX009506	JX010051	JX009805	JX010393
	F12PGSQ02	*M.* ×*domestica*	China	KF772127	KF772037	KF772097	KF772067	KF772157
	F10PGWFT2	*M.* ×*domestica*	China	KF772120	KF772030	KF772090	KF772060	KF772150
	F11PGQX26	*M.* ×*domestica*	China	KF772121	KF772031	KF772091	KF772061	KF772151
	F11PGLQ22	*M.* ×*domestica*	China	KF772122	KF772032	KF772092	KF772062	KF772152
	F12PGMJ01	*M.* ×*domestica*	China	KF772123	KF772033	KF772093	KF772063	KF772153
*C. simmondsii*	CBS 126524 ^2^	*Cyclamen* sp.	Netherlands	JQ948281	JQ949602	JQ948611	JQ948942	JQ949932
	CBS 122122 ^1^	*Carica papaya*	Australia	JQ948276	JQ949597	JQ948606	JQ948937	
*C. tropicale*	CBS 124949 ^1^	*Theobroma cacao*	Panama	JX010264	JX009489	JX010007	JX009870	
	ICMP 18672 ^3^	*Litchi chinensis*	Japan	JX010275	JX009480	JX010020	JX009826	

^1^ Cannon et al. (2012) [10]; ^2^ Damm et al. (2012a) [12]; ^3^ Weir et al. (2012) [52]; ^4^ Damm et al. (2012b) [53].

## 3. Results

### 3.1. Isolate Isolation

In total, 151 isolates were isolated from symptomatic leaf and fruit lesions in four apple-growing provinces. Among these, 98 were from bitter rot lesions, and 53 were from GLS lesions. Based on the conidial morphology and *ITS* sequence, 17 isolates were typical for the *C. acutatum* complex, while 134 isolates were typical for the *C. gloeosporioides* complex.

### 3.2. Phylogenetic Analysis

Based on *ITS* sequences and cultural characters, 32 representative isolates were chosen for further phylogenic analysis. The five-locus (*ITS*, *ACT*, *GAPDH*, *CHS*-1 and *TUB*2) phylogenetic analysis included 51 reference isolates [10,12,27]. Concatenated sequence alignment contained a total of 1916 characters, among which 551 were parsimony informative (28.8%). The BI tree, along with both the Bayesian posterior probability values and maximum likelihood bootstrap support values, are shown in Figure 1. The Bayesian tree was identical to the maximum likelihood tree in topology.

The phylogram supported eight defined clades, representing *C. aenigma*, *C. alienum*, *C. fructicola*, *C. gloeosporioides*, *C. nymphaeae, C. siamense* and a candidate for a new species, respectively. Four isolates clustered with *C. hymenocallidis* (CBS 125378), and one isolate grouped with *C. siamense sensu stricto* (CBS 130417 and ICMP 17795), which both belong to *C. siamense sensu lato* [52]. The clades of *C. fructicola* (CBS 130416), *C. aenigma* (ICMP 18608 and, ICMP 18686), *C. alienum* (ICMP 12071 and ICMP 18621) and *C. gloeosporioides* (CBS 112999 and CBS 119204) each included nine, three, one and six apple isolates, respectively.

**Figure 2:**
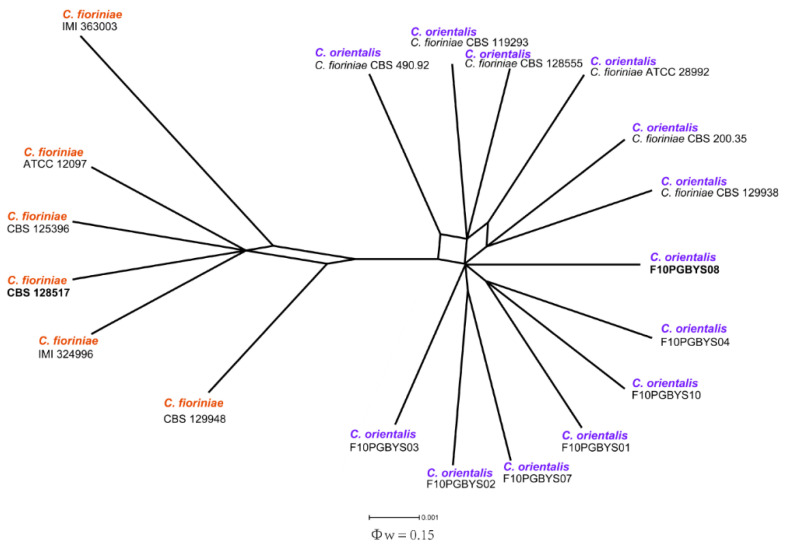
Pairwise homoplasy index (PHI) test of *C. fioriniae* and *C. orientalis* using both LogDet transformed and splits decomposition. PHI test results (Φ*_W_*) > 0.05 indicate the lack of recombination within the dataset.

The remaining eight isolates from the diseased apple fruits belonged to the *C. acutatum* complex. One isolate clustered together with *C. nymphaeae* (CBS 515.78 and IMI 370491), while the other seven formed a separate clade together with CBS 128555 (Figure 1). As revealed by the previous multi-locus molecular phylogenetic analysis [12], *C. fiorinae* contains two well-separated clades; one clade contains CBS 128555, and the other clade contains the type strain CBS 128517. Here, we propose that the CBS 12855 clade should better be defined as an independent taxon unit, which we have named *C. orientals*. The new species delimitation was also supported by the PHI analysis in which no obvious evidence of recombination was detected between the two clades (Figure 2).

### 3.3. Taxonomy

Based on the result of multigene phylogeny, the 32 *Colletotrichum* isolates characterized in this study were grouped into seven species: *C. aenigma* (three isolates), *C. alienum* (one isolate), *C. fructicola* (nine isolates), *C. gloeosporioides* (six isolates), *C. nymphaeae* (one isolate), *C. siamense* (five isolates) and *C. orientalis* (seven isolates).

**Figure 3:**
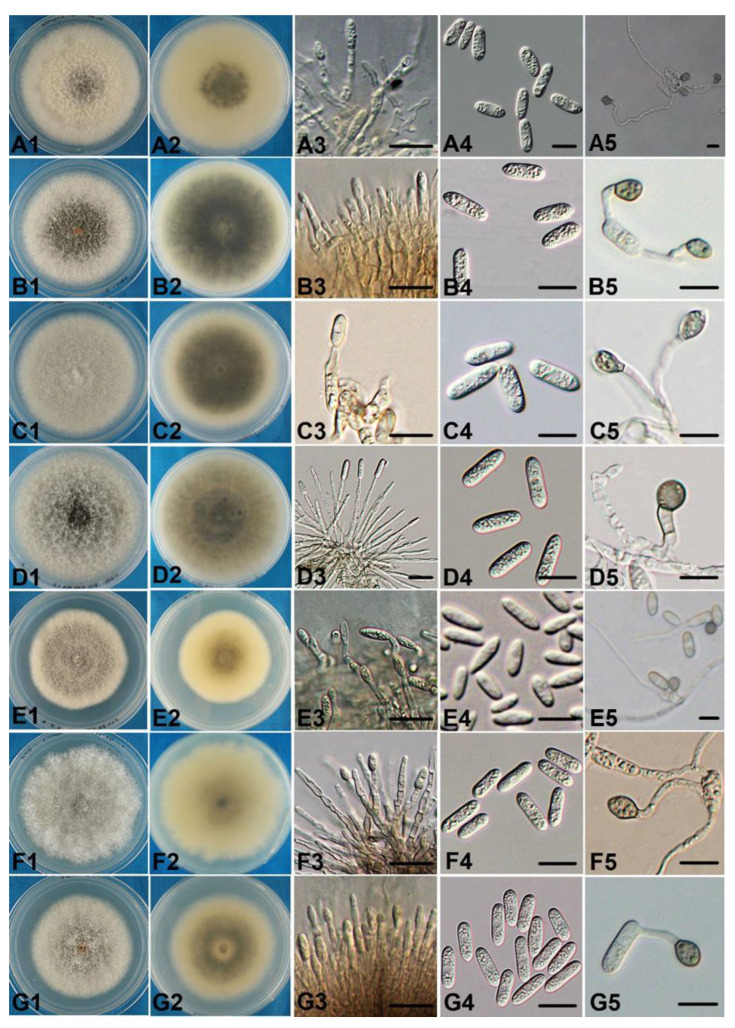
Morphological and cultural characters of *Colletotrichum* isolates: (**A**) *C. aenigma*; (**B**) *C. alienum*; (**C**) *C. fructicola*; (**D**) *C. gloeosporioides*; (**E**) *C. nymphaeae*; (**F**) *C. siamense*; (**G**) *C. hymenocallidis*. Upper (**1**) and reverse (**2**) of cultures on PDA; (**3**) conidiophores; (**4**) conidia; (**5**) appressoria. Bars = 10 μm.

***Colletotrichum aenigma*B. Weir & P.R. Johnst.** Studies in Mycology 73: 135. 2012. [52] Figure 3(A1–A5).

Description: *Vegetative hyphae* are 1–5.5 μm diam, hyaline, smooth-walled, septate and branched. *Conidiophores* are formed directly on hyphae. *Conidiophores* are hyaline and smooth-walled; they are sometimes septate and branched. *Conidiogenous cells* are hyaline, smooth-walled, cylindrical and not clearly separated from the hyphae by a septum. *Conidia* are straight, cylindric or clavate with rounded ends; sometimes they taper slightly to one end, (11.8–)15.5–17.5(–18.8) × (3.8–)4.5–5.5(–6) μm, mean ± SD = 16.46 ± 1.30 × 5.06 ± 0.56 μm (n = 50), L/W ratio = 3.3. *Appressoria* are elliptical or ovoid; some have broad, irregular lobes, (7.5–)8.5–9.5(–10.3) × (5.5–)6–7(–8.2) μm, mean ± SD = 9.07 ± 0.68 × 6.63 ± 0.50 μm (n = 50), L/W ratio = 1.4. *Colonies* on the PDA are flat with an entire margin; the aerial mycelium is sparse, cottony, and white-to-pale gray; in reverse, it is a pale honey and olivaceous gray towards the center with a growth rate of 68–80 mm in 7 d at 25 °C.

Specimen examined: China, Henan Province: Xiayi County, on the fruit surface of an apple (*Malus* ×*domestica* Borkh.), 7 September 2012, Dandan Fu, F12PGXY03; China, Henan Province: Xiayi County, on a leaf spot of an apple (*M.* ×*domestica* Borkh.), 7 September 2012, Wei Wang, W12PGYXY15.

Notes: *C. aenigma* could not be separated from *C. alienum* by *ITS* sequence analysis, nor from *C. tropicale* by *ACT*. The conidia of the holotype (ICMP 18608) of *C. aenigma* were (12–)14–15(–16.5) × (5–)6–6.5(–7.5) μm, and the appressoria were subglobose [52], whereas the isolate F12PGXY03 had longer and thinner conidia, and the appressoria were generally oval-shaped and longer than those of ICMP 18608. Additionally, the cultural characters of our isolates were different from those of ICMP 18608.

***Colletotrichumalienum*B. Weir & P.R. Johnst.** Studies in Mycology 73: 139. 2012. [52] Figure 3(B1–B5).

Description: *Vegetative hyphae* are 1–9 μm diam, hyaline, smooth-walled, septate and branched. *Conidiophores* are hyaline, smooth-walled, septate and branched. *Conidiogenous cells* are hyaline, smooth, ovoid-elliptical or short-cylindrical and often clearly have a septum. *Conidia* are straight, mostly cylindrical with broadly rounded ends; a few taper towards the basal end, (12.9–)15–17(–19.7) × (3.4–)4–5(–6.1) μm, mean ± SD = 16.27 ± 1.37 × 4.73 ± 0.56 μm (n = 50), L/W ratio = 3.4. *Appressoria* are mostly simple and subglobose or elliptical; a few have broad, irregular lobes, (6.8–)8–10(–10.8) × (5.1–)6–7(–7.6) μm, mean ± SD = 8.91 ± 0.98 × 6.46 ± 0.57 μm (n = 50), L/W ratio = 1.4. *Colonies* grown on the PDA (Difco) are 85 mm after 7 d at 25 °C; the aerial mycelium is dense, cottony and gray with an orange conidial ooze visible towards the center; in reverse, it is dark gray towards the center with sporadic black flecks and pale gray towards the edge.

Specimen examined: China, Henan Province: Zhengzhou City, on the fruit surface of an apple (*Malus* ×*domestica* Borkh.), 28 September 2011, Dandan Fu F11PGZH02.

***Colletotrichum fructicola*Prihastuti, L. Cai & K.D. Hyde.** Fungal Diversity 39: 96, 2009. [54] Figure 3(C1–C5).

Description: *Vegetative hyphae* are 1–11 µm diam, hyaline to pale brown, smooth-walled, septate and branched. *Conidiophores* are hyaline and smooth-walled; a few are septate and branched. *Conidiogenous cells* are hyaline, smooth, cylindrical and not clearly separated from the hyphae by a septum. *Conidia* are hyaline, aseptate, straight and cylindrical with both ends rounded or one end slightly acute, (13.1–)14.5–16(–18.5) × (4.5–)5–5.5(–6.2) µm, mean ± SD = 15.38 ± 1.16 × 5.29 ± 0.40 μm (n = 50), L/W ratio = 2.9. *Appressoria* are single or in loose groups, pale to dark brown, ovoid, cylindrical or fusoid and sometimes slightly irregular, (6–)8.5–11(–13) × (4.4–)5.5–6.5(–8.4) µm, mean ± SD = 9.66 ± 1.74 × 5.94 ± 0.81 μm (n = 50), L/W ratio = 1.6. *Colonies* on the PDA are 78*–*80 mm after 7 d. The aerial mycelium is white to pale gray, dense and cottony; in reverse, it is dark gray towards the center and pale gray at the edge.

Specimen examined: China, Henan Province: Shangqiu City, on the fruit surface of an apple (*Malus* ×*domestica* Borkh.), 6 September 2012, Dandan Fu F12PGSQ01, F12PGSQ05; from the leaf of an apple, Wei Wang, WW12PGYSQ06; Xiayi County, 7 September 2012, F12PGXY01. Liaoning Province: Xingcheng City, F10PGCJJ1, F10PGCJJ3; Huludao City, F10PGHLD1; Shandong Province: Yantai City, 29 September 2011, Dandan Fu, F11PGYT02, F11PGYT04.

Notes: The *ITS* sequence analysis did not separate *C. fructicola* from *C. aeschynomenes*, and the *ACT* sequence analysis could not separate it from *C. alienum*, *C. dianesei*, *C. queenslandicum* and *C. siamense*. Similarly, neither *GAPDH* nor *TUB*2 separated this species from *C. alienum*. The *CHS*-1 sequence analysis did not separate it from some of the isolates of *C. siamense*. Nevertheless, these taxa were well-distinguished using multi-gene analysis.

***Colletotrichum gloeosporioides*** (**Penz.**) **Penz. & Sacc. Atti Reale Ist. Veneto Sci.** **Lett. Arti., Series 6, 2: 670. 1884. [55]** Figure 3(D1–D5).

Description: *Vegetative hyaline* are 1–8 μm diam, hyaline to medium brown, smooth, septate and branched. *Conidiophores* are hyaline, smooth-walled, one to three celled and sometimes branched. *Conidiogenous cells* are hyaline, smooth, cylindrical and often clearly have a septum. *Conidia* are straight and mostly cylindrical with broadly rounded ends; they are sometimes slightly acute, tapering gradually to the ends, (13.1–)14–15(–16.1) × (3.8–)4.5–5.5(–5.8) μm, mean ± SD = 14.48 ± 0.70 × 5.12 ± 0.41 μm (n = 50), L/W ratio = 1.4. *Appressoria* are simple or in small groups and subglobose or elliptical; a few are irregular, (6.6–)7.5–9(–13.8) × (4.6–)5.5–6(–7.2) μm, mean ± SD = 8.32 ± 1.24 × 6.02± 0.72 μm (n = 50), L/W ratio = 1.4. *Colonies* grown on the PDA(Difco) are 75–80 mm after 7 d at 25 °C; the aerial mycelium is dense, cottony and pale gray to medium gray towards the center, in reverse, olivaceous gray, with sporadic dark gray flecks. Colonies on the OA are flat with an entire margin; the aerial mycelium is sparse, panniform and pale gray. An orange conidial ooze is visible in the mycelium.

Specimen examined: China, Shaanxi Province: Qian County, on the fruit surface of an apple (*Malus* ×*domestica* Borkh.), 24 September 2011, Dandan Fu F11PGQX17, 20 September 2010, F12PGLQ30, F12PGLQ33, F12PGLQ34; Dali County, 16 August 2012, F12PGDL01; Henan Province: Zhengzhou City, 28 September 2011, F11PGZH23.

***Colletotrichum nymphaeae*** (**Pass.**) **Aa. Netherlands J. PI. Pathol., Supplement 1 84: 110. 1978. [56]** Figure 3(E1–E5).

Description: *Vegetative hyphae* are 1–5 μm diam, hyaline, smooth, septate and branched. *Conidiophores* are formed directly on hyphae. *Conidiophores* are hyaline and smooth; a few are septate and branched. *Conidiogenous cells* are hyaline, smooth, cylindrical or fusiform and not clearly separated from the hyphae by a septum. *Conidia* are straight and cylindrical to clavate, with one end rounded and the other end or two ends acute, (6.8–)9–13(–15.9) × (3.4–)4–4.5(–5.3) μm, mean ± SD = 11.24 ± 2.19 × 4.24 ± 0.44 μm (n = 50), L/W ratio = 2.7. *Appressoria* are simple or in a small group and mostly subglobose or elliptical; a few have an irregular outline, (4.8–)6.5–7.5(–9) × (4.3–)5–6(–7.9) μm, mean ± SD = 6.97 ± 0.85 × 5.64 ± 0.60 μm (n = 50), L/W ratio = 1.2. *Colonies* on the PDA are flat with an entire margin. The aerial mycelium is sparse, grayish-yellow or cinnamon towards the center and white at the edge; in reverse, it is dark olivaceous gray. It has a growth rate of 54–60 mm after 7 d.

Specimen examined: China, Liaoning Province: Zhuanghe City, on the fruit surface of an apple (*Malus* ×*domestica* Borkh.), 20 September 2010, Jieli Zhuang F10PGBYS12.

***Colletotrichum siamense* Prihastuti, L. Cai & K. D. Hyde.** Fungal Diversity 39: 98. 2009. [54] Figure 3(F1–F5,G1–G5).

Description: *Vegetative hyphae* are 1–8 µm diam, hyaline to pale brown, smooth-walled, septate and branched. *Conidiophores* are hyaline to pale brown, smooth-walled and one or two celled; a few are branched. *Conidiogenous cells* are hyaline, smooth, cylindrical and clearly separated from the hyphae by a septum. *Conidia* are hyaline, aseptate, straight and cylindrical with both ends rounded, (11.9–)14.5–15.5(–18.9) × (4–)4.5–5(–5.5) µm, mean ± SD = 15.08 ± 1.14 × 4.87± 0.31 µm (n = 50), L/W ratio = 3.1. *Appressoria* are single or in loose groups, pale to dark brown, ovoid and subglobose or short, mean ± SD = 9.79 ± 1.60 × 5.94 ± 0.77 µm (n = 50), L/W ratio = 1.6. *Colonies* on the PDA have an entire margin. The aerial mycelium is white, and the colonies are dense, cottony, white to pale gray or dark gray. Occasionally, orange conidial ooze is visible in the center; in reverse, it is buff with sporadic dark gray spots or grayish dark towards the center and pale gray at the edge. Colonies on the PDA are 68*–*75 mm after 7 d.

Specimen examined: China, Henan Province: Shangqiu City, 6 September 2012, Dandan Fu F12PGSQ02; Mengjin County, 12 August 2012, Dandan Fu, F12PGMJ01; Shaanxi Province: Liquan County, on the fruit surface of an apple, 24 September 2011, Dandan Fu, F11PGLQ22; Qian County, F11PGQX26; Liaoning Province: Suizhong County, 20 September 2010, Jieli Zhuang F10PGWFT2.

Notes: After Prihastuti et al. separated *Colletotrichum* *siamense* from the *C. gloeosporioides* complex, more isolates from multiple hosts were identified as this species [54]. *ITS* sequences separated *C.* *siamense* well from other taxa, but the *ACT* sequence did not separate it from *C. alienum*, *C. hymenocallidis*, *C. queenslandicum* or *C. fructicola.* Similarly, *GAPDH* and *TUB*2 do not separate this species from *C. hymenocallidis*. *C. hymenocallidis* was first introduced by Yang et al. from *Hymenocallis americana* [57] but was recently synonymized with *C. siamense* [52]. However, Sharma et al. considered *C. siamense* to be a species complex based on an *ApMat* sequence analysis because *C. siamense* showed high sequence variability [58]. Moreover, Liu et al. indicated that more isolates need to be included to support further splitting of *C. siamense*, which possibly resurrects *C. hymenocallidis* [59].

**Figure 4:**
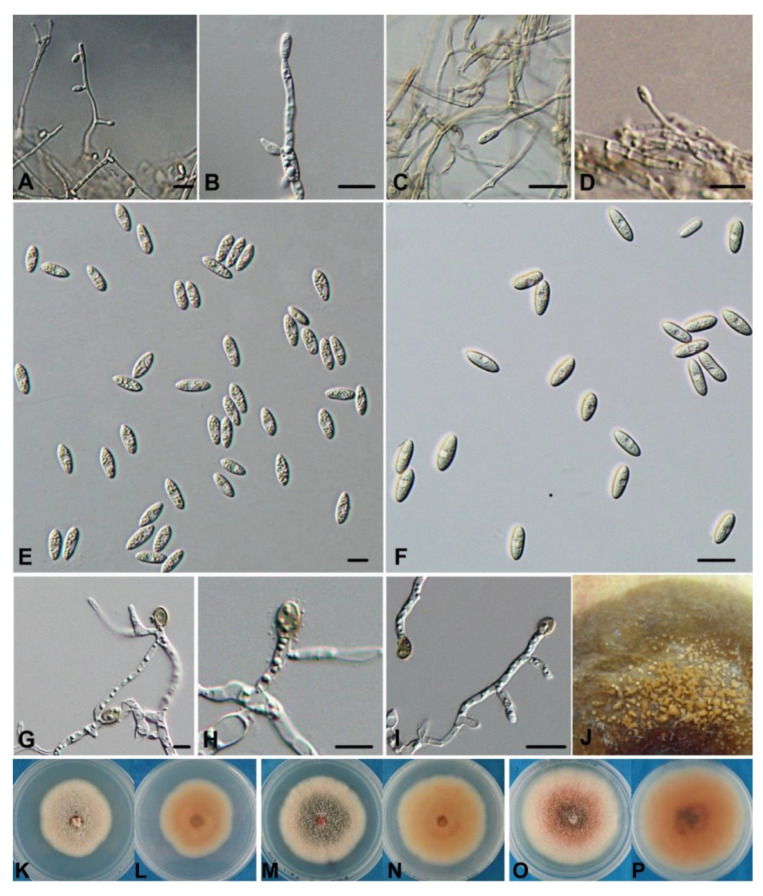
*Colletotrichum orientalis* (F10PGBYS08): (**A**–**D**) conidiophores; (**E**,**F**) conidia; (**G**–**I**) appressoria; (**J**) apple fruit lesion symptom with non-wounded conidial inoculation. Scale bars = 10 μm. (**K**,**L**) Colony on PDA (F10PGBYS08); (**M**,**N**) colony on PDA (F10PGBYS04); (**O**,**P**) colony on PDA (F10PGBYS05).

***Colletotrichum orientalis*Dandan Fu & G.Y. Sun, sp. nov.** Figure 4.

Mycobank: MB 808171.

Etymology: Referring to the isolates collected from the eastern region of China.

Description: *Vegetative hyphae* are 1–6 μm, hyaline, smooth-walled, septate and branched. *Conidiophores* are formed directly on the hyphae. *Conidiophores* are hyaline, smooth-walled, simple or septate and branched. *Conidia* are hyaline, smooth-walled, aseptate, straight and fusiform or cylindrical with both ends acute, (12.8–)14–16(–18.5) ×(3.9–)4–5(–5.5) μm, mean ± SD = 15.07 ± 1.23 × 4.51 ± 0.38 μm (n = 50), L/W ratio = 3.3 μm. *Appressoria* are single or in loose groups, pale-to-medium brown, smooth, oval-shaped and ellipsoidal or irregularly outlined, (7–)8–9.5(–11.5) × (4.4–)5.5–6(–7.2) μm, mean ± SD = 8.74 ± 0.99 × 5.84 ± 0.53 μm (n = 50), L/W ratio = 1.5. Colonies on the PDA have an entire margin and are compacted cottony to felty. They are orangish red towards the center and pale gray towards the edge. The aerial mycelium is white to pale gray, and the conidiomata are sparse with masses of orange conidia. In reverse pale brownish pink. Colonies on the OA have an entire margin. The aerial mycelium is sparse, white to pale gray, and on the surface with visible masses of orange conidia scattered in circles; in reverse, it is pale buff. Colonies on the PDA are 45*–*51 mm after 7 d (67*–*75 mm in 10 d).

Holotype: China*,* Liaoning Province: Zhuanghe City, on the fruit surface of an apple (*Malus* ×*domestica* Borkh.), 20 September 2010, Coll. Jieli Zhuang, F10PGBYS08 (CGMCC3.17216; isotype in HMAS244986 as dry culture).

Additional specimen examined: China, Liaoning Province: Zhuanghe City, on the fruit surface of an apple (*Malus* ×*domestica* Borkh.), 20 September 2010, Jieli Zhuang, F10PGBYS01 (CGMCC 3.17217), F10PGBYS02–04, F10PGBYS07–08, F10PGBYS10.

Notes: Freeman and Shabi studied isolates from fruit rot of apples and peaches (based on the *ITS* sequence, probably identifiable as *C. fioriniae*), which produced lesions on many different fruits, indicating that isolates of this group have the ability to cross-infect fruit from multiple hosts [60]. In this paper, we isolated seven isolates from apple bitter rot in Liaoning Province. A phylogenetic analysis (Figure 1) showed that they constituted a monophyletic clade together with the six *C. fioriniae* isolates (CBS 129938, CBS200.35, ATCC 28992, CBS 119293, CBS 128555 and CBS 490.92). In Damm et al. [12], the clade was well-separated from the clade containing the *C. fioriniae* holotype CBS 128517. Separation between the two clades was also evident in Damm et al. [12], which was treated as intraspecific heterogeneity. In this study, the PHI analysis detected no significant evidence of recombination between the two clades (Figure 2). Therefore, we denominate the clade containing the ABR isolates a new species.

### 3.4. Pathogenicity Tests

**Figure 5:**
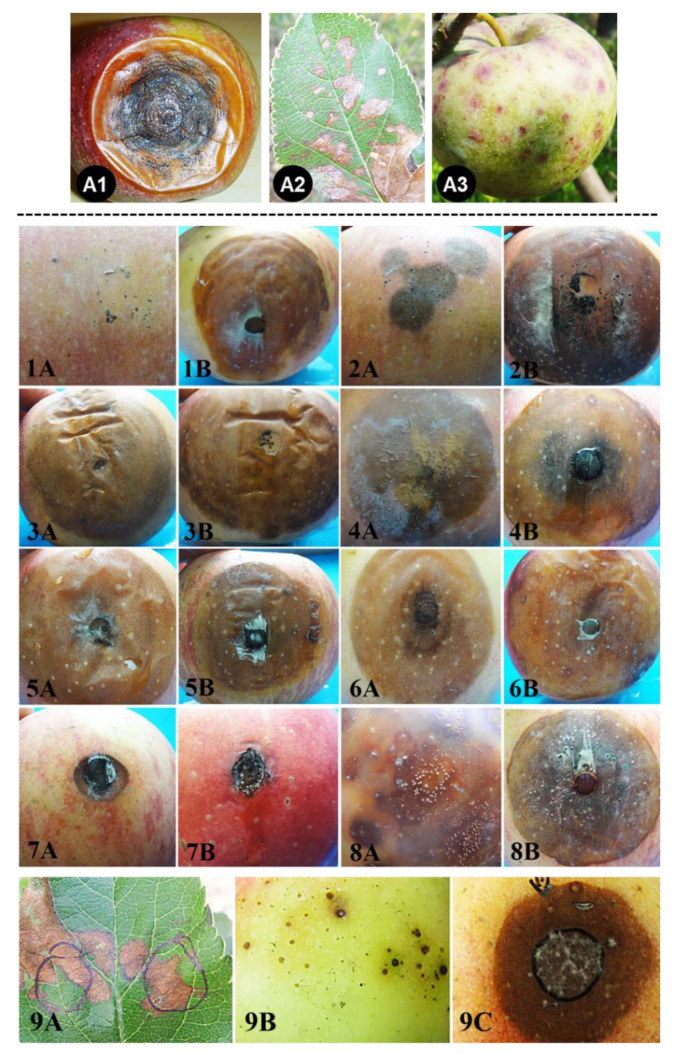
Typical field symptoms of ABR and GLS diseases (top) and artificial inoculation results (bottom). Top, field symptoms, (**A1**–**A3**) represent fruit ABR, GLS on leaves and fruits, respectively. Bottom, (**1A**–**8B**) represent typical symptoms on Fuji apples under unwounded or wounded inoculation conditions. **A**: Non-wounded; **B**: wounded; **1**: *C. aenigma* (F12PGXY03); **2**: *C. alienum* (F11PGZH02); **3**: *C. fructicola* (F12PGSQ01); **4**: *C. gloeosporioides* (F11PGQX17); **5**: *C. siamense* (F12PGSQ02); **6**: *C. siamense* (F11PGLQ22); **7**: *C. nymphaeae* (F10PGBYS12); **8**: *C. orientalis* (F10PGBYS08). (**9A**–**9C**) Symptoms on cv. Gala apple leaves and fruits inoculated with conidial suspension of isolate *C. fructicola* W12PGYSQ06 from GLS. (**9A**) Leaf inoculation; (**9B**) fruit from unwounded inoculation; (**9C**) fruit from wounded inoculation.

In the fruit infection assays, the isolates isolated from apples with bitter rot symptoms were pathogenic to the apple fruits in both the unwounded and wounded inoculations (Table 2). Dark brown rot lesions, similar in appearance, were produced in all cases (Figure 5). Of the non-wounded inoculations, *Colletotrichum alienum* (F11PGZH02) had the highest infection incidence (100%), whereas the isolates of *C. gloeosporioides* (F11PGQX17) and *C. orientalis* (F10PGBYS08) had the lowest incidences (33%); the others were in the middle. Of the wounded inoculations, all isolates had a 100% infection incidence. Lesions incurred by different isolates were similar in size, except that the lesions incurred by *C. nymphaeae* (F10PGBYS12, belonging to the *C. acutatum* complex) were apparently smaller (Figure 5).

Isolates isolated from the GLS lesions caused GLS lesions on both the apple fruits and leaves (Table 3). The isolates of *C. aenigma* (F12PGXY03, W12PGYXY15) and *C. fructicola* (F12PGSQ0503, W12PGYSQ06) were pathogenic on the leaves and fruits of Gala apples, but non-pathogenic on Fuji apple leaves or fruits in the non-wounded inoculation (Figure 5), which is in accordance with the observation that Fuji apples are resistant to GLS disease [61].

## 4. Discussion

China is the largest apple-producing country in the world. Bitter rot has been a common disease in almost all apple production areas and can cause large economic losses under disease-favorable temperature and humidity conditions. Glomerella leaf spot (GLS) has been a severe foliar disease on cvs. Gala, Jonagold and Golden Delicious in the USA and Brazil. It was found first in Henan, China, in 2010 [28]. Now, it has become prevalent in all major apple-producing areas in China. Thus far, however, the species diversity of apple *Colletotrichum* pathogens in China is largely unclear. In this study, we collected and characterized 151 isolates from four apple-producing provinces and identified six known species, as well as one new species, demonstrating that diverse *Colletotrichum* species can infect apples. Moreover, *C. orientalis* was shown for the first time to be an apple *Colletotrichum* pathogen.

Among the identified species, *C. siamense*, *C. fructicola*, *C. aenigma*, *C. alienum* and *C. gloeosporioides* belong to the CGSC, while *C. orientalis* and *C. nymphaeae* belong to the CASC. Overall, the CGSC species appear to be more prevalent compared with the CASC species. Moreover, fruit isolates and leaf isolates differ significantly in their genetic makeups. Seven species were recognized as isolates from apple fruits, whereas only two (*C. fructicola* and *C. aenigma*) were recognized as leaf spot isolates. In a pathogenicity test, ABR isolates fail to incur GLS symptoms, and the GLS isolates fail to incur ABR symptoms, indicating a pathogenic differentiation among the two groups of pathogens. Such results are in accordance with a previous study that demonstrated the intraspecific differentiation in the pathogenicity of GLS and ABR for *C. fructicola* [62].

*C. siamense* is a species that includes members from diverse hosts and that has a worldwide distribution. The diversity is so high that there has been controversy regarding whether it should be treated as a single species or a species complex. In a recent study carried out by Liu and others [63], six independent species very close to *C. siamense s. str.* (*C. communis*, *C. hymenocallidis*, *C. dianesei*, *C. endomangiferae*, *C. jasmini-sambac* and *C. murrayae*) were renamed as *C. siamense*. In this study, the four characterized isolates clustered together with *C. hymenocallidis*, and one isolate clustered with *C. siamense s. str*. Based on broad species criteria, these isolates should all be regarded as *C. siamense sensus lato*. *C. fructicola* represents another important pathogen species identified in this study. *C. fructicola* has a very broad host range, having been isolated from over eight plant families as endophytes and as plant pathogens. In this study, *C. fructicola* was isolated from both bitter rot and Glomerella leaf spot lesions. *C. fructicola* causes Glomerella leaf spot in Brazil but has been more commonly identified as a bitter rot pathogen in central USA, Brazil and Uruguay. In Uruguay in particular, most isolates from apple bitter rot were identified as *C. fructicola*. Interestingly, despite the prevalence of *C. fructicola* in Uruguay, Glomerella leaf spot disease does not occur in the field [62]. In the future, it would be interesting to determine whether there are distinctive *C. fructicola* populations for isolates from leaf lesions and fruit lesions in China.

In a previous study [12], *C. fiorinae* has been defined as a species with two well-separated clades. We propose here that the two clades should be regarded as different species due to the fact that the pairwise homoplasy index (PHI) analysis in SplitsTree did not detect evidence of recombination between them. Therefore, we have named the new lineage *C. orientalis*. *C. alienum* and *C. gloeosporioides* are two other species common in fruits. Interestingly, *C. alienum* has only been reported in New Zealand and Australia, and *C. gloeosporioides* has never been reported on apples in China. The identification of these species on apples in China highlights the importance of a diversity survey.

Compared with apple bitter rot, GLS is a relatively new disease. Velho and others have suggested that GLS pathogens originate from apple bitter rot pathogens [62]. In this study, all isolates caused fruit rot upon wound inoculation, whereas only the isolates from the leaf spot lesions incurred leaf spot symptoms. Importantly, such pathogenic differentiation could occur in the same species (e.g., F12PGSQ01 and W12PGYSQ06). Such a pathogenicity differentiation pattern is in line with the report by Velho and others [62]. Isolates showing an intraspecific pathogenic variation would be valuable resources for comparative studies aiming to dissect the mechanisms that underlie the adaptive evolution of apple *Colletotrichum* pathogens.

In summary, based on a systemic survey of *Colletotrichum* isolates, in this study, we identified seven species associated with the GLS and ABR diseases in China, highlighting the rich species diversity of *Colletotrichum* spp. on apples. There are tens of apple-producing provinces in China that are variable in terms of climate and soil conditions; further survey efforts into the hidden species diversity and the structural variations among the different regions is critical for effective control of these two important diseases.

## Figures and Tables

**Table 2:** Pathogenicity test of representative *Colletotrichum* isolates on Fuji apple fruits.

Species	Isolate	Non-Wounded	Wounded
*C. alienum*	F11PGZH02	+++	+++
*C. fructicola*	F12PGSQ01	++	+++
*C. gloeosporioides*	F11PGQX17	+	+++
*C. nymphaeae*	F10PGBYS12	++	+++
*C. siamense*	F12PGSQ02	++	+++
*C. orientalis*	F10PGBYS08	+	+++

+: Infection incidence < 50%; ++: 50% < Infection incidence < 100%; +++: Infection incidence = 100%.

**Table 3:** Pathogenicity test of selected isolates on apple leaves.

Species	Isolate	Origin	Inoculation Cultivar	Inoculation Outcome
*C. aenigma*	F12PGXY03	GLS lesion	Fuji	−
			Gala	+
	W12PGYXY15	GLS lesion	Fuji	−
			Gala	+
*C. fructicola*	F12PGSQ05	GLS lesion	Fuji	−
			Gala	+
	W12PGYSQ06	GLS lesion	Fuji	−
			Gala	+
*C. alienum*	F11PGZH02	ABR lesion	Fuji	−
			Gala	−
*C. fructicola*	F12PGSQ01	ABR lesion	Fuji	−
			Gala	−
*C. gloeosporioides*	F11PGQX17	ABR lesion	Fuji	−
			Gala	−
*C. nymphaeae*	F10PGBYS12	ABR lesion	Fuji	−
			Gala	−
*C. siamense*	F12PGSQ02	ABR lesion	Fuji	−
			Gala	−
*C. orientalis*	F10PGBYS08	ABR lesion	Fuji	−
			Gala	−

+: Pathogenic; −: Non-pathogenic.

## Data Availability

Not applicable.
